# Digital assessment in myasthenia gravis: evidence, validation, and a proposed framework for autoimmune heterogeneity, remote monitoring, and clinical endpoints

**DOI:** 10.3389/fimmu.2026.1948530

**Published:** 2026-09-03

**Authors:** Mengyi Wang, Yanpeng Huang, Zhibo Chai, Zhaoqing Li, Wenjun Qiao, Weifeng Xie

**Affiliations:** 1 The First Clinical College, Liaoning University of Traditional Chinese Medicine, Shenyang, China; 2 Affiliated Hospital of Liaoning University of Traditional Chinese Medicine, Shenyang, China

**Keywords:** autoimmunity, digital endpoint, digital health technology, electronic patient-reported outcome, myasthenia gravis, remote monitoring, speech analysis, wearable sensor

## Abstract

Myasthenia gravis (MG) is an antibody-mediated autoimmune disorder of the neuromuscular junction in which weakness fluctuates across symptom domains, daily activities, and treatment cycles. Established measures such as the Quantitative Myasthenia Gravis score, Myasthenia Gravis Composite, Myasthenia Gravis Activities of Daily Living scale, and MG-specific quality-of-life instruments remain the principal clinical anchors, but intermittent assessments may miss intraday variability, exertional fatigability, and changes across treatment cycles. This structured narrative review examines electronic patient-reported outcomes, smartphone-based active tasks, speech and video analysis, wearable sensing, telemedicine services, prediction models, and candidate digital endpoints. It also distinguishes the validation requirements of these modalities and critically appraises the MG-specific evidence. Most published studies demonstrate feasibility, adherence, acquisition reliability, or preliminary construct validity; most are small, short, and incompletely stratified by antibody subtype, and independent external validation and impact studies remain uncommon. Accordingly, subtype-specific digital trajectories should be regarded as a research hypothesis rather than as an established feature of AChR-, MuSK-, LRP4-associated, or seronegative MG. We present an MG digital phenotype and a five-stage closed-loop workflow as organizing frameworks, not as consensus standards or validated care pathways. Near-term value is most plausible in structured remote follow-up, treatment-cycle characterization, and exploratory trial measurement, provided that outputs remain domain-specific, respiratory safety is protected, and abnormal signals undergo human clinical review.

## 1 Introduction

Myasthenia gravis (MG) is an acquired autoimmune disease of the neuromuscular junction. Acetylcholine receptor (AChR) antibodies commonly impair transmission through receptor blockade, receptor loss, and complement-mediated postsynaptic injury, whereas muscle-specific kinase (MuSK) antibodies mainly disrupt agrin-LRP4-MuSK signaling and receptor clustering. LRP4-associated and seronegative MG add further biological and clinical heterogeneity (1–3). Despite these differences, fluctuating fatigable weakness across ocular, bulbar, axial, limb, and respiratory domains is the shared clinical expression; severe deterioration may culminate in myasthenic crisis (1, 4, 5).

A single clinic observation cannot reconstruct the full disease course. Weakness changes with repeated activity, rest, infection, medication timing, and treatment exposure, while a rested examination may underrepresent impairment during meals, prolonged speech, work, or late-day activity. The clinically relevant phenotype is therefore longitudinal: it includes fluctuation amplitude, activity-induced decline, recovery after rest, and the onset or waning of treatment response (5–10).

Clinical care and therapeutic trials nevertheless depend on standardized instruments. The QMG, MGC, MG-ADL, and MG-QOL15r provide validated and interpretable anchors, although their sampling density, recall windows, and domain sensitivity differ (11–17). In this review, digital assessment is treated as an extension of these measures, not a substitute for neurological examination, objective respiratory assessment, or clinical judgment.

### 1.1 Review approach

We conducted a structured narrative search of PubMed/MEDLINE, Crossref-indexed records, relevant journal and publisher websites, and primary regulatory websites through 6 August 2026. Search concepts combined “myasthenia gravis” with digital health, telemedicine, remote monitoring, smartphone, wearable, accelerometry, speech, voice, video, computer vision, eye movement, electronic patient-reported outcome, digital endpoint, digital biomarker, prediction, artificial intelligence, and validation. English-language peer-reviewed articles were prioritized; protocols were retained when they clarified platform design or cohort provenance, and recent records were checked by forward and backward citation tracking. MG-specific evidence was prioritized for claims about clinical performance. General digital-health, clinical-outcome-assessment, regulatory, and medical-AI literature was used only when MG-specific methodological evidence was absent.

Multiple reports arising from the same platform or cohort were treated as related evidence rather than independent replication. In particular, the MyaLink protocol, feasibility study, symptom-variability analysis, and prediction study describe successive questions within one research program (8, 10, 16, 18). Study design, sample size, follow-up, validation status, external validation, clinical action, funding or developer involvement, and major limitations were extracted for principal MG-specific studies (**Table 1**). Because no protocol was registered and no formal risk-of-bias instrument was applied, this review is vulnerable to selective retrieval and interpretive judgment; the study-level table and explicit evidence categories are intended to make those limitations visible.

**Table 1:** Study-level synthesis of principal MG-specific digital-assessment evidence.

Study/program	Design, setting, follow-up	N	Modality/platform	Serology/treatment context	Anchor/principal finding	Validation status/external validation	Care change/impact	Funding or developer involvement	Key limitations/cohort relation
Steyaert et al., 2023 (45)	Decentralized prospective observational, United States, 3 months	113 enrolled; 82 completed	Daily Smartphone ePROs with optional passive smartphone/wearable activity data	Serology not reported in performance analyses; heterogeneous routine care	Remote repeated data collection was feasible and captured longitudinal symptoms	Feasibility/data completeness; no independent external validation	No impact evaluation	Industry/platform involvement reported	Attrition; no clinical-action test; independent from MyaLink
Stein et al., 2025 (16)	Single-center prospective feasibility, 3 months	30	MyaLink app, PROMs, wearable, home digital spirometry, clinician portal	Predominantly AChR-positive; mixed therapies; no powered subtype analysis	High adherence and usability; home data transmission feasible	Feasibility/usability only; no external validation	Clinicians could review/adjust care, but outcome impact was not tested	Academic-industry software partnership and grant support reported	Small sample; overlapping MyaLink intervention cohort
Stein et al., 2026 symptom variability (18)	Randomized controlled program; 12-week descriptive intervention analysis	45 total; 30 intervention	MyaLink multimodal monitoring and messaging	AChR-predominant; pooled analyses	Greater score variability and more interventions in participants with hospitalization/exacerbation	Exploratory association; no external validation	Treatment adjustments occurred; causal outcome benefit not established	Same platform/developer program	*Post hoc* subgrouping; overlapping MyaLink cohort
Stein et al., 2026 prediction (10)	Model development within 12-week MyaLink intervention cohort	30	PROMs, patient-performed FVC/SBCT, wearables; machine-learning classifier	20 AChR-positive, 1 MuSK-positive; no subtype performance analysis	AUROC 0.85; sensitivity 0.65-0.82 at 1–2 false alarms/patient/week	Leave-one-subject-out internal CV; no independent external validation	No prospective impact evaluation	Qurasoft platform support; industry grants reported without study-design involvement	Small event set; imputation and false-alarm burden; same MyaLink cohort
Mishra et al., 2025 (17)	Feasibility study	20	BioDigit MG: tablet speech/facial tasks, ePROs, activity/posture	Subtype-stratified performance not reported	Feasibility and acceptability of multimodal acquisition	Feasibility; no external clinical validation	No impact study	Developer involvement disclosed in article	Small sample; short feasibility focus
Garbey et al., 2024 (27)	Clinic-based digital MG core-exam study	51 MG; 15 controls	Video/audio active tasks and AI-assisted quantification	Subtype-specific performance not established	Identified feasibility and acquisition limitations	Early analytical/construct evaluation; no independent external validation	No care-impact study	Academic development program	Acquisition constraints and limited sample diversity
Garbey et al., 2026 (58)	Repeated examinations across six centers	52	Quantitative telemedicine MG core examination	Subtype analyses not reported	Instructions and video quality affected reliability	Multicenter reliability assessment; not external clinical validation of outcomes	No impact study	Academic MGNet-related development	Repeated videos but limited longitudinal clinical change
Vesperinas et al., 2026 (19)	Single-center prospective observational pilot; 15-month follow-up	31	Weekly app-based MG-ADL monitoring	Generalized MG; subtype-specific accuracy not established	Detected more exacerbations than routine visits and informed some treatment adjustments	Prospective detection study; no external validation	No evidence of improved long-term outcomes	Funding disclosed in article	Small single-center cohort; no randomized impact comparison
Zanghi et al., 2026 (52)	Single-center cross-sectional clinic recording	17	Connected-speech acoustic analysis	Subtype-stratified results limited or absent	Acoustic features differed despite stable global scores	Cross-sectional construct exploration	No care-impact study	Not presented as a commercial platform validation	Not longitudinal or remote; very small sample
Norling et al., 2026 DIG-MG (59)	Randomized controlled feasibility/lifestyle study; 6-week baseline plus 12-week intervention and 6-week follow-up	81 randomized; 72 completed	Smart ring, digital phenotyping, lifestyle intervention	Mixed MG; not powered for antibody-stratified effects	High feasibility/adherence; no significant MG-ADL or fatigue improvement versus control	Randomized feasibility/effect estimate; no external replication	No demonstrated clinical benefit on primary symptom outcomes	Funding and device relationships disclosed in article	Digitally selected, mildly affected cohort; ceiling effects; no objective MG performance measures

The table distinguishes feasibility, measurement validation, prediction, clinical action, and outcome impact. Related MyaLink publications are explicitly treated as an overlapping research program rather than independent replication. NR, not reported; PROM, patient-reported outcome measure; FVC, forced vital capacity; SBCT, single-breath count test; CV, cross-validation; AUROC, area under the receiver operating characteristic curve.

## 2 Immunological and clinical rationale for digital assessment

### 2.1 Fluctuation as a measurable feature of MG

MG is particularly suited to longitudinal digital assessment because weakness varies over minutes, hours, and days and is modified by activity and treatment state (12, 19). Ocular, bulbar, limb, or respiratory symptoms may change after sleep, repeated use, emotional stress, infection, or medication adjustment. A clinic examination captures only one physiological state and may be influenced by acetylcholinesterase inhibitor timing, procedural variation, and interrater differences (12, 19, 20). Home monitoring has identified episodes of worsening that were not documented during scheduled visits, illustrating that the value of digital measurement lies in reconstructing disease course rather than merely increasing the quantity of data (19).

### 2.2 Autoimmune heterogeneity and treatment-cycle dynamics

AChR-MG, MuSK-MG, LRP4-associated MG, and seronegative disease differ in pathogenic mechanisms, dominant symptom patterns, thymic associations, prognosis, and treatment response (1–3). These are established clinical and immunological differences. By contrast, the proposition that each subtype has a reproducible digital trajectory remains largely untested: most MG digital studies are small, AChR-predominant, or report pooled performance without antibody-stratified accuracy, responsiveness, adherence, or missingness analyses.

Treatment context may plausibly shape longitudinal signals. Complement inhibition, FcRn blockade, B-cell-directed therapy, corticosteroids, conventional immunosuppression, rescue treatment, and dose timing can produce different onset, plateau, and waning patterns (21–25). High-frequency assessment may characterize those phases, but it cannot infer the immune mechanism from behavior or sensor data alone. Antibody status, treatment class, dose timing, infection, rescue therapy, and objective respiratory findings therefore remain contextual variables rather than labels predicted by the digital signal. **Table 2** separates demonstrated evidence from hypotheses and unstudied questions.

**Table 2:** Autoimmune heterogeneity and candidate digital trajectories: evidence boundaries.

Serological/treatment context	Established clinical or immunological features	Plausible digital implication (hypothesis)	Actual subtype-specific digital evidence	Evidence status
AChR-positive MG; complement/FcRn-targeted or conventional therapy	Most common subtype; complement-mediated injury is prominent; broad ocular, bulbar, limb, and respiratory involvement (1, 2)	Treatment-cycle changes may appear in domain-specific ePRO, activity, speech, or video trajectories	Most digital cohorts are AChR-predominant, but formal antibody-stratified performance and responsiveness are rarely reported	Demonstrated recruitment; subtype-specific digital performance largely not studied
MuSK-positive MG; B-cell/FcRn-directed or other therapy	Often greater bulbar, facial, neck, and respiratory involvement; different immunopathology and treatment response (1, 2)	Bulbar speech/video and respiratory trajectories may be particularly relevant; device burden may differ	Very small numbers in published digital cohorts; no adequately powered subtype validation	Hypothesized; not demonstrated
LRP4-associated MG	Less common and heterogeneous; diagnostic and clinical boundaries remain under study (3)	Ocular/generalized domain mix may require individualized modality selection	No convincing LRP4-stratified digital validation identified	Not studied
Seronegative MG	Heterogeneous group requiring careful diagnostic characterization (3, 117)	Greater diagnostic uncertainty may increase the need for clinical anchoring and decrease specificity of digital signals	No seronegative-specific performance, responsiveness, or missingness analysis identified	Not studied
Any subtype during targeted or cyclic treatment	Onset, plateau, and waning differ by mechanism and dosing schedule (21–25)	High-frequency trajectories may characterize treatment response windows	Digital studies rarely test drug-class-specific validity or interaction with antibody subtype	Plausible; insufficient direct evidence

“Demonstrated” indicates direct evidence in the cited MG digital literature; “hypothesized” indicates a biologically or clinically plausible relationship that has not been validated; “not studied” indicates that the review did not identify adequate subtype-specific evidence by the search date.

### 2.3 Domain-specific measurement

MG does not produce a single homogeneous deficit. Ptosis, diplopia, dysarthria, dysphagia, chewing fatigue, head drop, impaired mobility, dyspnea, and generalized fatigue arise from different physiological functions and are not optimally measured by one technology (26). Video is suited to eyelid and ocular-motor performance; speech tasks can probe bulbar and respiratory–phonatory fatigability; wearables can describe activity bouts, postural transitions, and recovery; and ePROs capture the patient’s functional experience (17, 27, 28). Multimodal systems should therefore retain interpretable ocular, bulbar, limb, respiratory, and generalized functional outputs rather than collapsing all data into an opaque global score.

### 2.4 Ecological validity and patient relevance

Ecological validity concerns whether an assessment reflects function in ordinary life (29). Commuting, working, reading, speaking, eating, climbing stairs, and household tasks can expose burdens that are minimized during a brief, rested examination (30, 31). Active tasks, passive sensing, and patient-generated reports can sample symptoms in these contexts and define their timing, triggers, and recovery (19, 29, 32). Ecological validity, however, is not achieved simply by collecting data at home. A measure is clinically useful only when its output is linked to a patient-relevant function, a validated clinical anchor, and a decision pathway (29, 32–35).

## 3 Conventional outcome measures as clinical anchors

### 3.1 Established outcome measures

Standardized outcome measures support clinical stratification, treatment evaluation, and endpoint selection. MG-ADL records patient-perceived limitations in ocular, bulbar, respiratory, and limb function; QMG emphasizes clinician-administered strength and fatigability tasks; MGC combines clinician- and patient-reported information; MG-QOL15r evaluates disease-related quality of life; the Myasthenia Gravis Impairment Index (MGII) integrates perceived impairment with examination findings; and the Myasthenia Gravis Foundation of America Post-Intervention Status (MGFA-PIS) classifies overall post-treatment status (11–14, 36–38). Together, these instruments provide the common language required to interpret new digital measures.

### 3.2 Strengths and limitations

Clinician-administered measures provide structured procedures and established interpretive frameworks. QMG covers ocular, facial, bulbar, limb, neck, and respiratory function, while MGC reduces testing burden by combining selected high-value items (11, 13). Patient-reported measures contribute information that strength testing cannot capture. MG-ADL is widely used in contemporary trials, including studies of batoclimab, efgartigimod, ravulizumab, and rozanolixizumab, and MG-QOL15r captures broader daily-life consequences (14, 21–24, 36). These measures are indispensable because their validity and clinical meaning are comparatively well established.

Their limitations include rater variability, procedural heterogeneity, incomplete domain coverage, floor or ceiling effects, and low temporal resolution (11, 12, 39, 40). Test performance may vary with equipment, posture, instructions, medication timing, and patient effort. Moreover, MG-ADL, QMG, and MG-QOL15r measure different constructs, and no single scale captures the complete phenotype. Patient-reported respiratory items cannot replace spirometry or other objective testing when respiratory involvement is suspected (41). Scheduled visits and retrospective recall also provide limited information about peaks, troughs, activity-induced weakness, and recovery after rest.

### 3.3 Complementarity with digital measures

Digital measures should extend the temporal and contextual reach of conventional scales. MG-ADL, QMG, MGC, and MG-QOL15r define status at standardized time points; ePROs, speech and video tasks, and wearable sensing can show how that status changes within a day, after exertion, or across a treatment cycle (29, 32–34). A stronger design pairs the two forms of measurement: a digital feature is interpreted against an established domain-specific anchor, while the digital time series supplies information that the anchor cannot provide. This approach preserves clinical meaning and permits evaluation of incremental value.

## 4 Digital health technologies for MG

### 4.1 Terminology and measurement classes

In this review, a digital health technology (DHT) is the hardware-software system used to collect, transmit, process, or display health information. A digital measure is the quantified output generated by that system. An electronic clinical outcome assessment (eCOA) is an electronically administered clinician-, observer-, performance-, or patient-reported assessment; an ePRO is the patient-reported subset. A sensor-derived measure is calculated from a physical signal captured by a sensor. A digital biomarker is a digitally measured indicator of a biological or pathogenic process or a response to an intervention and is not used here as an umbrella term for every app output. A predictive or decision-support model estimates a future or current clinical state to inform, but not replace, human judgment. A digital endpoint is a precisely defined trial variable derived from digital data. “Digital phenotype” refers to a longitudinal representation assembled from multiple digital observations; the MG-specific version proposed in Section 9 is an authors’ conceptual synthesis (33, 34, 42, 43).

These categories serve different purposes. ePROs and diaries capture patient experience; active speech, video, or movement tasks apply standardized provocation; passive sensors characterize behavior or physiology in daily life; prediction models combine inputs to estimate an outcome; and telemedicine services organize communication and clinical workflow. Each class therefore requires a different evidentiary pathway (Section 5 and **Table 3**).

**Table 3:** Modality-specific evidence requirements for MG digital assessment.

Technology/intended use	Core validity question	Minimum evidence before stronger clinical claims	Additional requirements for deployment or trial use
ePRO/eCOA	Does the electronic assessment measure the intended patient or clinical construct?	Content validity; scoring; reliability; construct validity; responsiveness; meaningful change; recall period; electronic implementation and mode equivalence where relevant; accessibility and data integrity (47, 72–75)	Prespecified schedule; missing-data plan; language/accessibility testing; audit trail; interpretation of change
Sensor-derived measure	Does the system accurately capture a signal and convert it into a valid MG-relevant feature?	Technical verification; repeatability and reproducibility; analytical robustness; domain-matched clinical validation; independent external dataset (33, 34, 76, 79)	Device/version control; intended-environment testing; usability; subgroup and multidevice performance
Predictive/decision-support model	Does the model predict a defined outcome over a defined horizon with clinically acceptable error?	Clear outcome/horizon; leakage prevention; appropriate internal validation; discrimination and calibration; false-alarm burden; external validation and transportability (71, 80, 81)	Prospective impact evaluation; human review; threshold governance; monitoring for performance drift
Telemedicine service/multimodal platform	Can the service be used safely, sustainably, and effectively in its workflow?	Usability; adherence; role allocation; data-review process; safety and escalation; interoperability; implementation fidelity (85, 86, 88, 89)	Clinical-outcome or resource-use evaluation; reimbursement and sustainability; contingency plan for outages and missed data
Digital clinical-trial endpoint	Is the derived variable fit for its specified role in the estimand and interpretable as meaningful change?	Concept of interest; endpoint definition; acquisition schedule; aggregation; valid-data rules; missingness; reliability/responsiveness; meaningful within-patient change (75, 90–92)	Locked versions; sensitivity analyses; auditable change control; early regulatory dialog; context-specific acceptability
Cross-cutting governance	Can the technology be used equitably and accountably?	Privacy and security; informed consent; representative datasets; fairness assessment; accessible alternatives; human factors (93–97)	Post-deployment monitoring; incident response; model/update governance; transparent limitations

This table is the authors’ synthesis of the cited literature and regulatory principles. It is not a consensus standard, and the evidence threshold must be proportional to the intended use and risk. DHT, digital health technology; eCOA, electronic clinical outcome assessment; ePRO, electronic patient-reported outcome.

### 4.2 Electronic patient-reported outcomes and diaries

ePROs and electronic diaries are among the most readily deployable approaches to digital MG monitoring. Electronic MG-ADL assessments can be completed daily, weekly, or according to a treatment cycle. Symptom diaries can additionally record the onset, duration, and fluctuation of ocular, bulbar, limb, and respiratory manifestations together with medication use, dose changes, missed doses, rescue therapy, infection, physical exertion, and sleep. Some platforms also track treatment-related adverse events, healthcare use, and work impairment, allowing symptoms to be temporally linked to treatment exposure and daily context (44). Electronic symptom reports can distinguish self-reported worsening from nonworsening periods and capture changes between scheduled visits (45), while longer digital studies can collect longitudinal information on disease burden, treatment, and quality of life (46). These tools are less costly and easier to deploy across regions than dedicated sensors or complex algorithms and directly capture the patient experience (18). Their validity nevertheless depends on comprehension, adherence, and recall, and total MG-ADL scores may conceal divergent changes across symptom domains (47).

Electronic administration increases sampling frequency but does not remove subjectivity, recall effects, response shift, or burden. Longer recall periods can emphasize the most recent or most severe episode, whereas frequent prompts increase delayed entries, missingness, and attrition (45, 48). Missing data may be nonrandom, particularly in patients with greater disease burden, limited digital skills, visual impairment, or lower engagement. Interface complexity, authentication, notification frequency, accessibility, and the perceived usefulness of feedback influence sustained participation.

Older adults and people with visual impairment, reduced fine motor control, cognitive limitations, or restricted internet access may require simplified interfaces, training, caregiver support, or nondigital alternatives (49). ePROs also cannot independently distinguish MG activity from mood, comorbidity, infection, or ordinary physical exertion. Individual MG-ADL items show different relationships with examination findings and quality-of-life constructs (47). ePRO data should therefore be analyzed at both total-score and domain levels and time-aligned with QMG, MGC, respiratory testing, or objective digital measures.

### 4.3 Speech and voice assessment

Bulbar MG may cause imprecise articulation, reduced intensity, vocal instability, hypernasality, and progressive deterioration during sustained speech. Tasks such as prolonged phonation, counting, rapid syllable repetition, standardized reading, and free conversation impose different loads on velopharyngeal closure, laryngeal function, respiratory support, and orofacial movement (50, 51). Candidate features include maximum phonation time, fundamental frequency and its variability, intensity, jitter, shimmer, harmonic-to-noise ratio, speech and articulation rates, syllable duration, pause proportion, and nasality. Segment-by-segment analysis can estimate the slope of deterioration, change from the beginning to the end of the task, maximum decline, and recovery after rest.

Connected-speech studies indicate that intensity, rate, and articulation rhythm may differ even when a global clinical score is stable, suggesting partly distinct constructs (50, 52). This evidence requires careful qualification: the 2026 connected-speech study was a small, single-center, cross-sectional clinic study of 17 patients, not validation of longitudinal remote speech monitoring. Recording is feasible with common microphones, but device characteristics, noise, language, accent, age, sex, and respiratory comorbidity affect acoustic values. Current evidence supports exploratory within-patient use more strongly than stand-alone diagnosis or treatment decisions.

### 4.4 Video and computer-vision assessment

Ocular MG is highly task-dependent and may shift between muscles and gaze directions. Standardized smartphone or tablet video can measure palpebral fissure height, bilateral asymmetry, progressive eyelid descent after sustained upgaze, blink patterns, and selected facial movements (28, 53, 54). Eye-movement recordings can derive saccade amplitude, velocity, latency, fixation drift, binocular misalignment, and decrements during repeated saccades or smooth pursuit (55–57). Routine video is appropriate for coarse eyelid and facial assessment, whereas precise ocular alignment or saccadic metrics require calibration targets, fixed geometry, adequate frame rates, or dedicated video-oculography.

Acquisition conditions are a major source of error. Backlighting, camera angle, head rotation, compression, bandwidth, reflections from glasses, and inconsistent targets can alter derived measurements (27, 28). In the multicenter repeated-examination study of 52 patients, variations in examiner instruction and video quality materially affected reliability (58). Small development datasets may also underrepresent age, sex, skin tone, ethnicity, and facial morphology. Standardized instructions, automated quality checks, explicit annotation rules, expert review of critical labels, multidevice testing, and independent external validation are therefore prerequisites for broader claims.

### 4.5 Wearable and respiratory sensing

Wearable devices can record activity, sedentary time, sleep, posture, and selected cardiorespiratory signals (17, 59–61). In MG, activity-bout duration, interruptions, postural transitions, and recovery may be more informative than a generic daily step total. These features remain nonspecific and are influenced by occupation, mood, pain, cardiopulmonary disease, sleep disorders, weather, and device wear. Within-person interpretation is generally more defensible than direct inference from group averages (59–63).

Respiratory data require a separate safety boundary. Formal respiratory assessment by trained clinicians, patient-performed home FVC or single-breath counting, cough-related measures, and nonspecific wearable pulse or oxygen-saturation signals are different measurement classes. MyaLink demonstrated that trained patients could transmit home spirometry data over three months, but it did not establish full analytical or clinical validation for detecting deterioration or crisis (16). Cough peak flow and office-based respiratory measures have supporting reliability or clinical data in their specific settings (64, 65), while the MG-ADL breathing item has limited value as a stand-alone substitute for spirometry (41). None of these findings validates all home respiratory outputs as interchangeable MG endpoints.

### 4.6 Multimodal platforms and telemedicine

BioDigit MG combines tablet-based speech and facial tasks with MG-ADL, MG-QOL15r, activity, and posture data; its published study involved 20 participants and primarily established feasibility (17). The remote MG core examination covers ptosis, diplopia, facial strength, dysarthria, single-breath counting, arm endurance, and sit-to-stand tasks; a study of 51 patients and 15 controls highlighted acquisition constraints, and a subsequent six-center study of 52 patients showed that instructions and video quality affected reliability (27, 58, 66). MyaLink integrates an app, clinician portal, PROMs, wearables, home pulmonary measurements, and messaging; its publications address protocol, feasibility, variability, and prediction within an overlapping program rather than four independent replications (8, 10, 16, 18).

Feasibility and data transfer do not establish construct validity, prediction, clinical impact, or improved outcomes. The DIG-MG randomized trial enrolled 81 participants, with 72 completing the study; its 6-week baseline, 12-week intervention, and 6-week follow-up supported an intention-to-treat analysis and did not demonstrate significant improvement in MG-ADL or fatigue versus control (59). Platforms are most interpretable when they use the smallest complementary set of measures required for a stated purpose, retain domain-level outputs, and define who reviews the data and what action follows. **Table 4** summarizes the digital approaches discussed throughout Section 4 by mapping MG symptom domains to candidate measures, clinical anchors, key limitations, and current evidence maturity.

**Table 4:** Mapping MG symptom domains to candidate digital measures, clinical anchors, and evidence maturity.

MG domain	Candidate approach	Candidate outputs	Key limitations/safety boundary	Clinical anchors	Current evidence status
Ocular	Standardized smartphone video; calibrated video-oculography	Palpebral fissure height, eyelid descent, blink pattern, saccade metrics, binocular alignment (28, 53–57)	Lighting, camera geometry, gaze calibration, frame rate, device and facial diversity	Ocular examination; QMG/MGC ocular items; MG-ADL ptosis and diplopia items	Feasibility and selected measurement studies; limited external validation and no regulatory-ready MG endpoint
Bulbar	Sustained phonation, counting, reading, connected speech, facial video	Phonation time, intensity, articulation rate, pause structure, nasality, within-task decline (27, 50–52)	Microphone and noise; language/accent; respiratory support; small cross-sectional datasets	QMG/MGC bulbar items; MG-ADL speech, chewing, swallowing	Exploratory feature and feasibility evidence; longitudinal responsiveness not established
Limb/axial	Accelerometry, inertial sensors, arm endurance, sit-to-stand	Activity-bout duration, interruptions, transitions, asymmetry, fatigue slope (17, 58, 60–62, 67)	Lifestyle, occupation, pain, mood, placement; weak disease specificity	QMG limb items; MGC; clinician-observed functional tasks	Cross-sectional and limited longitudinal association; no validated action threshold
Respiratory	Formal testing; trained patient-performed home FVC/SBCT; cough measures; contextual pulse/SpO2	FVC trend, single-breath count, cough peak flow, respiratory-phonatory endurance (16, 41, 64–66)	Technique, posture, calibration and comorbidity. Home or wearable values cannot exclude important weakness or replace urgent assessment	Formal respiratory assessment and clinical examination; MG-ADL breathing item only as supplementary report	Home collection feasible in a small study; device-, protocol-, and context-specific clinical validation remains incomplete
General function/treatment cycle	ePROs, diaries, multimodal platforms	Daily/weekly MG-ADL, fluctuation burden, response onset, waning trajectory (18, 19, 45, 59)	Recall, response effects, adherence, nonrandom missingness, overlapping cohorts	MG-ADL, QMG, MGC, MG-QOL15r; treatment and rescue records	Feasibility and detection of additional episodes shown; improved long-term outcomes not demonstrated

Entries list candidate outputs, not equivalently validated endpoints. Evidence status summarizes the present MG literature and does not imply regulatory qualification. MG, myasthenia gravis; ePRO, electronic patient-reported outcome; FVC, forced vital capacity; MGC, Myasthenia Gravis Composite; MG-ADL, Myasthenia Gravis Activities of Daily Living; QMG, Quantitative Myasthenia Gravis; SBCT, single-breath count test; SpO2, peripheral oxygen saturation.

## 5 Modality-specific validation and evidence requirements

### 5.1 Context of use and evidence boundaries

Validation starts with a defined context of use: target population, concept of interest, setting, user, acquisition schedule, output, clinical or trial role, and consequence of error. The evidence package depends on the technology class and risk. The V3 and V3+ frameworks provide a strong basis for sensor-based DHTs, but they do not make ePROs, predictive models, telemedicine services, and trial endpoints methodologically identical (33, 34). **Table 3** is therefore an authors’ synthesis of published methodological and regulatory principles, not an internationally accepted MG validation standard.

We distinguish feasibility, data-transfer or acquisition reliability, analytical performance, construct or clinical validity, prediction, clinical action, and improved outcomes. A study can satisfy one level without satisfying the next. The current MG literature is concentrated in the earlier levels, as summarized in **Table 1**. Methodological appraisals of MG AI research and broader medical AI likewise identify incomplete reporting, limited robustness assessment, and insufficient external validation as barriers to translation (68–71).

### 5.2 eCOA and ePRO validation

For an ePRO or other eCOA, the central question is whether the instrument captures the intended patient or clinical construct in the target population and whether electronic implementation preserves its interpretation. Relevant evidence includes content validity, scoring, reliability, construct validity, responsiveness, meaningful within-patient change, recall period, language and accessibility, data integrity, and mode equivalence when an established paper instrument is migrated electronically (47, 48, 72–75). Hardware sensor verification is not the principal framework unless the assessment also depends on a sensor.

### 5.3 Sensor-derived measures

Sensor-derived speech, video, activity, sleep, or respiratory measures require technical verification of signal capture, analytical validation of the feature-extraction pipeline, clinical validation against a domain-matched construct, and usability testing in the intended environment (33, 34, 76–79). Repeatability, reproducibility across devices and sites, robustness to noise and missing data, locked preprocessing, and independent datasets are particularly important. A feature validated for one task, device, or setting cannot automatically be transferred to another.

### 5.4 Predictive and decision-support models

Prediction models require additional safeguards: explicit outcome and prediction-horizon definitions, separation of development and evaluation data, prevention of information leakage, appropriate internal validation, calibration, clinically interpretable sensitivity and false-alarm burden, independent external validation, transportability assessment, and prospective evaluation of whether use improves decisions or outcomes (69, 71, 80, 81). Cross-validation within a small cohort estimates internal performance; it is not external validation. A model with acceptable AUROC may still be unusable if false alerts overwhelm clinicians or if performance shifts across devices, centers, or serological subgroups.

### 5.5 Telemedicine services and multimodal platforms

A telemedicine service is evaluated as a care process, not only as a measurement device. Evidence must address workflow ownership, accessibility, patient and clinician burden, data review, safety, escalation, interoperability, cybersecurity, implementation fidelity, and clinical outcomes. Multimodal platforms also need traceability: the contribution and failure mode of each component should remain visible rather than being hidden in a single composite score (82–89).

### 5.6 Digital endpoints in trials

A digital endpoint requires a prespecified concept of interest and a defined role in the estimand. The protocol and statistical analysis plan should state acquisition timing, aggregation windows, valid-data rules, missing-data handling, treatment of intercurrent events, version control, and interpretation of meaningful within-patient change (75, 90–92). A weekly MG-ADL or QMG assessment is not automatically a digital endpoint; the mode of acquisition, derivation, and endpoint definition must themselves be specified. Regulatory qualification is one route, but a measure may also be accepted within a particular drug-development program after context-specific discussion and evidence review.

### 5.7 Cross-cutting usability, fairness, and governance

All modalities require accessible interfaces, representative development and validation samples, transparent handling of missingness, privacy protection, cybersecurity, version control, and human-factor testing. Subgroup evaluation should be planned where sample size permits, but sparse strata must be reported as indeterminate rather than interpreted from unstable estimates. Human-centered clinical AI places clinician-patient interaction, explainability, and responsibility for action at the center of deployment (43, 70, 85, 93, 94).

## 6 Clinical applications

### 6.1 Remote follow-up

Remote follow-up is most defensible for clinically stable patients with an established diagnosis and treatment plan who do not require a complex or urgent examination. Periodic ePROs, structured video tasks, and carefully selected home measurements can help teams decide whether remote management remains appropriate. The MG TeleScore corresponded substantially with face-to-face measures, although task execution and posture influenced fatigability assessment (98). Weekly home MG-ADL monitoring in a 31-patient single-center pilot detected more worsening episodes than routine visits and contributed to some treatment adjustments, but it did not establish reduced health-care use or improved long-term outcomes (19).

Remote respiratory monitoring is restricted to stable patients who have been trained in the device and protocol and who have a defined clinical contact and escalation plan. New or progressive dyspnea, rapidly worsening swallowing or speech, weak cough, rapidly generalizing weakness, inability to complete a previously reliable task, or concerning discordance between symptoms and home values warrants immediate clinical contact or emergency in-person assessment rather than repeated home measurement (5, 41, 65).

### 6.2 Detection of clinical worsening

Multimodal monitoring may provide an early signal when several domain-specific changes occur relative to an individual baseline. However, pulse, oxygen saturation, activity, and sleep are nonspecific, and an alert is not a diagnosis. In the 30-patient MyaLink model-development study, leave-one-subject-out cross-validation produced an AUROC of 0.85 and sensitivity of 0.65-0.82 when one to two false alarms per patient per week were accepted (10). The study had no independent external validation or prospective impact evaluation. Its results therefore support hypothesis generation and clinical-review triggers, not autonomous crisis prediction.

### 6.3 Monitoring treatment response

Longitudinal assessment can describe symptom fluctuation and recovery after treatment, but validity is treatment- and modality-specific. Sampling frequency should match the expected pharmacodynamic timescale: acetylcholinesterase inhibitors may require dense pre- and post-dose observations, whereas corticosteroids and conventional immunosuppressants are evaluated over weeks to months (99). Fixed weekly MG-ADL or QMG assessments in trials of complement or FcRn-targeted therapies help describe onset and maintenance, but they are not digital endpoints unless digitally acquired and prespecified as such (22, 23). Home MG-ADL monitoring may identify recurrence between efgartigimod cycles after clinical confirmation (19). Stand-alone digital efficacy endpoints for plasma exchange, intravenous immunoglobulin, or targeted therapies have not been validated (100, 101).

### 6.4 Long-term individualized management

Longitudinal digital data are most informative when interpreted within the same patient over time. Platforms can align MG-ADL or other ePROs, speech endurance, activity, sleep, environmental temperature, and medication use to identify patterns related to heat, sleep deprivation, specific activities, or afternoon and evening worsening (18, 102). Comparing symptoms and function before and after activity and measuring time to recovery may characterize individual fatigue and recovery curves and estimate tolerable activity intensity and duration. Uniform step or exercise targets are inappropriate because group-level relationships among activity, sleep, fatigue, and symptom severity may not apply to a specific individual and may even differ in direction (61). Accelerometry has shown lower high-intensity activity and longer sedentary time in stable MG, but activity is not linearly related to disease severity and cannot by itself define a safe threshold (60). During cyclic therapy, home assessment may help estimate an individual treatment-response window (59). Such information can support personalized follow-up and lifestyle counseling but must be clinically verified and should not automatically generate exercise prescriptions or treatment changes.

### 6.5 Clinical boundaries and escalation

Digital tools provide supplementary information and risk screening; they cannot replace acute respiratory assessment, specialist judgment, or management of myasthenic crisis. Progressive dyspnea, rapidly worsening swallowing or speech, a weak cough, rapidly generalizing weakness, or respiratory failure requires immediate in-person evaluation and objective respiratory testing (5). FVC, maximal inspiratory pressure, or sniff nasal inspiratory pressure may be abnormal despite apparently mild symptoms, so a patient report, single-breath count, home spirometry value, pulse, or SpO2 cannot exclude respiratory involvement (41, 65).

A system intended for clinical use needs prospectively defined alert levels, repeat-testing rules where safe, nurse or physician review, response times, coverage outside working hours, handling of missing data, emergency referral, documentation, and patient communication. The platform must state clearly that it is not a continuously staffed emergency service. Escalation pathways developed in other remote-monitoring programs require MG-specific validation before adoption (103).

## 7 Digital endpoints in clinical trials

### 7.1 Rationale in the era of targeted immunotherapy

Targeted complement inhibitors, FcRn antagonists, and B-cell-directed therapies have increased interest in outcomes that are sensitive to change and treatment-cycle dynamics. Trials of ravulizumab, batoclimab, and inebilizumab used MG-ADL or QMG as central outcomes (21, 23, 25). Conventional scales remain the efficacy foundation; digitally acquired home ePRO, speech, activity, or functional data may supplement them by characterizing fluctuation, time to sustained improvement, or recovery of real-world activity. Such candidate variables remain exploratory until their acquisition, derivation, validity, and interpretation are prespecified.

### 7.2 Uses across the trial lifecycle

Digital measures may support several stages of an MG trial. A short digital run-in period can repeatedly collect MG-ADL or other ePROs, speech, activity, and home-based functional measures to characterize baseline variability, identify highly fluctuating phenotypes, and improve randomization or stratification (18, 45). After randomization, high-frequency time-series data may estimate response slopes, time to clinical response, and within-cycle variability and may detect early signals between fixed visits. Patients who worsen or require hospitalization show greater MG-ADL variability, and weekly monitoring can identify changes not recorded in routine care (18, 19). Electronic clinical outcome assessments, wearables, and video visits may decentralize selected procedures, broaden participation, and reduce unnecessary site visits, but they cannot replace safety assessments or standardized neurological examination (104). Patient organizations can improve study design by helping reduce task burden and clarify the purpose of data collection, thereby supporting recruitment and long-term engagement. Longitudinal digital cohorts can also link treatment exposure, symptoms, healthcare use, and quality of life and contribute to real-world evidence generation (46, 105).

### 7.3 Evidence requirements for registrational use

For a primary or key secondary digital endpoint, reliability, analytical accuracy where applicable, sensitivity to clinical change, and interpretability must be demonstrated in the target MG population and context of use. Meaningful within-patient change should be estimated with patient- or clinician-anchored methods rather than imported from a conventional scale (74, 75). Protocols and statistical analysis plans need valid-data criteria, aggregation windows, missing-data definitions, imputation and sensitivity analyses, version control, and bridging validation after material changes (90–92). Regulatory qualification is not the only pathway: a measure may be considered within a specific development program after early dialog and context-specific evidence review. We did not identify evidence that an MG digital measure has broad, cross-program regulatory acceptance as of the search date; this is a scoped literature and regulatory-source observation, not proof of absence in every jurisdiction or confidential program.

## 8 Ethical, equity, and implementation considerations

### 8.1 Data privacy and security

Videos, speech recordings, and continuous activity trajectories can reveal facial identity, the home environment, routines, and behavioral patterns. Even after direct identifiers are removed, longitudinal sensor data may remain re-identifiable (95). Informed consent should therefore support genuine understanding and specify which third parties may access which data and for what purpose (96, 97). Systems should follow data-minimization principles and collect only raw signals or derived features required for the intended clinical purpose. Encryption in transit and at rest, role-based access, least-privilege permissions, and auditable logs should be implemented. Data-sharing agreements should define authorized users, scope and duration of access, and patient control (106). Cloud services and cross-border processing require attention to secondary use, data controller responsibilities, storage jurisdiction, and applicable regulation. De-identification reduces but does not eliminate the risk associated with continuous sensing and cannot replace ethical and legal oversight (107).

### 8.2 Equity, fairness, and the digital divide

Older age, visual or hearing impairment, cognitive limitations, impaired fine motor control, limited digital experience, language barriers, and unequal access to devices or connectivity may restrict participation (108–110). Digital readiness should be assessed before enrollment, and training, caregiver support, telephone options, accessible design, or nondigital alternatives should be available. Technology-related exclusion should not be mislabeled as poor adherence. Missingness itself may be a marker of access, disability, or burden and should be analyzed rather than simply imputed away.

Fairness also depends on dataset composition, labeling, device performance, and workflow. Homogeneous development data can reduce generalizability and perpetuate inequity (93, 94). Performance should be reported across clinically relevant demographic and technical strata when sample sizes support stable estimates. Antibody-stratified MG performance is particularly sparse; pooled results must not be extrapolated to small MuSK-, LRP4-associated, or seronegative groups. Where subgroup samples are inadequate, the correct conclusion is “not established,” not equivalence or inferiority. Participatory design with patients, caregivers, nurses, and clinicians can expose burdens or failure modes that accuracy metrics miss (43, 85).

### 8.3 Workflow, interoperability, and sustainability

For routine care, usefulness depends on whether clinicians can act on the data. Remote monitoring creates responsibilities for data review, patient communication, and technical support. Nonspecific or nonactionable thresholds may generate excessive alerts and reduce clinical responsiveness (88, 111). Programs should specify monitoring periods, first-line reviewers, escalation levels, response times, handling of missed data, and emergency referral responsibilities and should make clear that the platform is not a continuously staffed emergency service. Systems that are separate from the electronic health record create duplicate log-ins and fragmented information. Although integration is technically feasible, evidence of clinical benefit remains limited, and standardized application programming interfaces may be constrained by cost, insufficient real-world testing, or missing key data elements (89, 112). Reimbursement, staffing, device replacement, cybersecurity maintenance, and data migration after vendor withdrawal must be included in life-cycle planning. Uncertain workflow ownership, coding, payment, and program sustainability continue to limit adoption and equitable access (113, 114).

## 9 Future directions

### 9.1 A proposed MG digital phenotype

We propose “MG digital phenotype” as a research construct: an individualized longitudinal representation that aligns ocular, bulbar, limb, respiratory, and generalized-function trajectories with ePROs, active tasks, passive sensing, medication exposure, treatment cycles, infection, and clinically verified events. This extends general digital-phenotyping concepts but is not an accepted endpoint, consensus recommendation, or validated predictor (42, 115). Domain-specific trajectories should remain visible; compressing all inputs into an opaque global score risks hiding acquisition error, confounding, and clinically discordant domains (82, 83, 116). BioDigit MG and DIG-MG demonstrate technical feasibility of multimodal acquisition, not independent prediction of crisis or improved long-term outcomes (17, 59).

### 9.2 Autoimmune heterogeneity as a research agenda

The proposed framework links digital trajectories to serological phenotype and treatment context without implying that digital data identify antibody status. Future studies could test whether AChR-, MuSK-, LRP4-associated, and seronegative MG differ in domain fluctuation, adherence, sensor performance, missingness, or response trajectories. At present, these are biologically plausible questions grounded in established phenotype and treatment differences (1–3, 117), but direct subtype-specific digital evidence is insufficient.

### 9.3 Research priorities and a proposed five-stage closed-loop workflow

Priority studies are preregistered, multicenter, longitudinal, and designed around a specified clinical question. Acquisition protocols, device versions, calibration, time synchronization, quality control, and missing-data procedures need to be harmonized, while external datasets remain independent of model development (80, 81, 118). Samples should reflect age, sex, race or ethnicity, language, digital literacy, geography, device platform, disease severity, and serological subtype. Models for worsening or treatment response need an explicit outcome, prediction horizon, action threshold, calibration assessment, false-alarm burden, and incremental value beyond MG-ADL, QMG, MGC, and prior trajectory.

**Figure 1:**
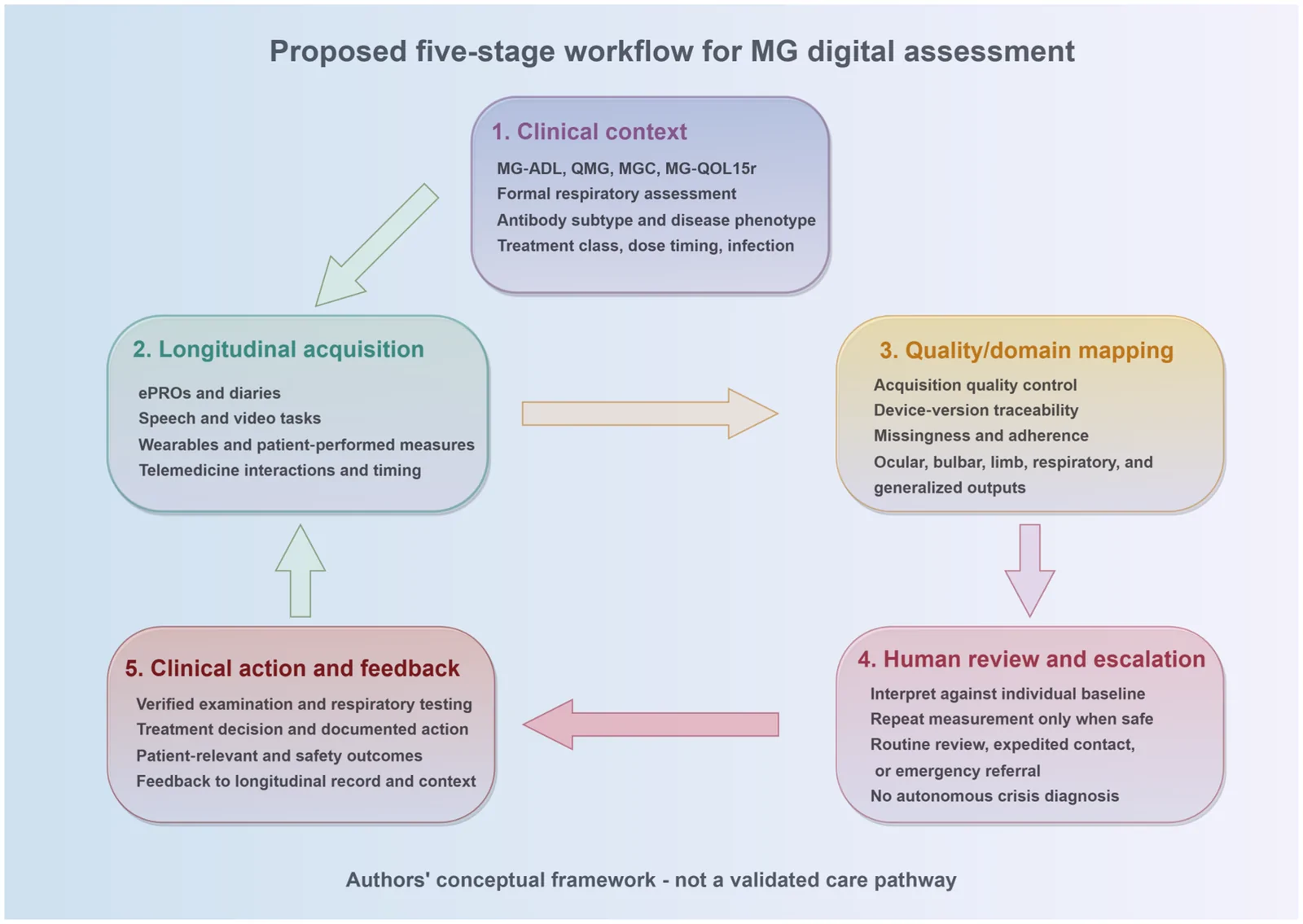
Proposed five-stage closed-loop workflow for MG digital assessment. The sequence is clinical context →longitudinal acquisition →quality/domain mapping→ human review and escalation →clinical action and feedback. Verified outcomes feed back into the longitudinal record and clinical context. The figure is an authors’ conceptual framework and has not been validated as a care pathway. Created with Figdraw.

**Figure 1** presents a proposed five-stage closed-loop workflow. Stage 1 establishes the clinical context, including clinical anchors, antibody status, treatment exposure, and respiratory findings. Stage 2 acquires longitudinal observations, followed by Stage 3 quality control and domain mapping. In Stage 4, human reviewers interpret abnormal patterns and determine whether repeat measurement, routine follow-up, expedited assessment, or emergency escalation is appropriate. Stage 5 documents verified examination findings, clinical actions, and patient-relevant outcomes; these verified outcomes feed back into the longitudinal record and clinical context. This is an organizing framework for research and workflow design, not a validated care pathway.

## 10 Discussion

Digital assessment can sample MG fluctuation, exertional fatigability, real-world function, and treatment-cycle dynamics more frequently than conventional visits. The evidence, however, is uneven. ePRO studies mainly show feasibility and denser symptom sampling; speech, video, and wearable studies largely establish candidate features or acquisition reliability; the first prediction models rely on small overlapping cohorts; and telemedicine platforms have not yet shown consistent improvement in long-term clinical outcomes. **Table 1** makes these levels explicit rather than treating every positive study as equivalent validation.

Methodological quality remains the principal constraint. Small samples, single-center recruitment, short follow-up, variable acquisition, incomplete subgroup reporting, internal rather than external validation, and untested alert thresholds limit generalizability (10, 16, 17, 19, 27, 58, 59, 68). Multimodal models may broaden domain coverage but can also accumulate measurement error, obscure modality-specific failure, and increase dimensionality (82, 83, 116). Robustness and uncertainty need to be reported alongside average performance (69).

The review’s conceptual contribution is deliberately separated from its evidence synthesis. The MG digital phenotype, autoimmune-context framework, modality-specific validation map, and five-stage closed-loop workflow organize research questions; they are not consensus standards. Their added value over general digital-biomarker and human-centered AI frameworks lies in MG-specific domain mapping, fluctuation, respiratory safety, treatment-cycle timing, and explicit separation of established subtype biology from untested digital hypotheses (33, 42, 43, 68).

Near-term clinical use is most plausible when the question is narrow: identifying stable patients who need earlier review, characterizing symptom recurrence within a treatment cycle, or supplementing exploratory trial outcomes. Each use requires its own acquisition schedule, clinical anchors, missing-data rules, action thresholds, and evidence standard. The aim is a parsimonious, interpretable set of measures that adds information beyond established assessment without weakening safety or equity.

## 11 Conclusion

Digital technologies can extend MG assessment from intermittent observations to longitudinal, context-sensitive measurement, but the present evidence base is dominated by feasibility and exploratory validation. ePROs, active speech and video tasks, wearables, home measurements, prediction models, and telemedicine services require distinct validation pathways. Respiratory signals remain supplementary and cannot exclude clinically important weakness or replace urgent in-person assessment. Antibody subtype and treatment context are essential interpretive variables, yet subtype-specific digital performance remains largely unstudied. The proposed MG digital phenotype and five-stage closed-loop workflow should be tested as research frameworks rather than adopted as standards. Progress will require transparent evidence identification, multicenter external validation, prespecified clinical use, representative samples, interpretable domain-level outputs, human review, and evidence that digital measurement improves decisions or outcomes.
